# The Application of Transcutaneous CO_2_ Pressure Monitoring in the Anesthesia of Obese Patients Undergoing Laparoscopic Bariatric Surgery

**DOI:** 10.1371/journal.pone.0091563

**Published:** 2014-04-03

**Authors:** Shijiang Liu, Jie Sun, Xing Chen, Yingying Yu, Xuan Liu, Cunming Liu

**Affiliations:** 1 Department of Anesthesiology, The First Affiliated Hospital of Nanjing Medical University, Nanjing, China; 2 Department of Anesthesiology, The First Affiliated Hospital of Nanjing Medical University, Nanjing, China; 3 Department of Project Management, Jiangsu New Energy Development Company, Jiangsu Guoxin Investment Group, Nanjing, China; 4 Department of Anesthesiology, The First Affiliated Hospital of Nanjing Medical University, Nanjing, China; 5 Department of Anesthesiology, General Hospital of TISCO, TaiYuan, China; 6 Department of Anesthesiology, The First Affiliated Hospital of Nanjing Medical University, Nanjing, China; Massachusetts General Hospital, United States of America

## Abstract

To investigate the correlation and accuracy of transcutaneous carbon dioxide partial pressure (P_TC_CO_2_) with regard to arterial carbon dioxide partial pressure (P_a_CO_2_) in severe obese patients undergoing laparoscopic bariatric surgery. Twenty-one patients with BMI>35 kg/m^2^ were enrolled in our study. Their P_a_CO_2_, end-tidal carbon dioxide partial pressure (P_et_CO_2_), as well as P_TC_CO_2_ values were measured at before pneumoperitoneum and 30 min, 60 min, 120 min after pneumoperitoneum respectively. Then the differences between each pair of values (P_et_CO_2_–P_a_CO_2_) and_._ (P_TC_CO_2_–P_a_CO_2)_ were calculated. Bland–Altman method, correlation and regression analysis, as well as exact probability method and two way contingency table were employed for the data analysis. 21 adults (aged 19–54 yr, mean 29, SD 9 yr; weight 86–160 kg, mean119.3, SD 22.1 kg; BMI 35.3–51.1 kg/m^2^, mean 42.1,SD 5.4 kg/m^2^) were finally included in this study. One patient was eliminated due to the use of vaso-excitor material phenylephrine during anesthesia induction. Eighty-four sample sets were obtained. The average P_a_CO_2_–P_TC_CO_2_ difference was 0.9±1.3 mmHg (mean±SD). And the average P_a_CO_2_–P_et_CO_2_ difference was 10.3±2.3 mmHg (mean±SD). The linear regression equation of P_a_CO_2_–P_et_CO_2_ is P_et_CO_2_ = 11.58+0.57×P_a_CO_2_ (r^2^ = 0.64, P<0.01), whereas the one of P_a_CO_2_–P_TC_CO_2_ is P_TC_CO_2_ = 0.60+0.97×P_a_CO_2_ (r^2^ = 0.89). The LOA (limits of agreement) of 95% average P_a_CO_2_–P_et_CO_2_ difference is 10.3±4.6 mmHg (mean±1.96 SD), while the LOA of 95% average P_a_CO_2_–P_TC_CO2 difference is 0.9±2.6 mmHg (mean±1.96 SD). In conclusion, transcutaneous carbon dioxide monitoring provides a better estimate of PaCO_2_ than P_et_CO_2_ in severe obese patients undergoing laparoscopic bariatric surgery.

## Introduction

Currently the “gold standard” technique for the measurement of arterial carbon dioxide partial pressure (P_a_CO_2_) is performed by direct analysis of arterial blood gases (ABG), but this method is invasive, intermittent and may cause iatrogenic anemia in infants. The end-tidal carbon dioxide partial pressure (P_et_CO_2_) measurement has been widely used for the continuous noninvasive monitoring of carbon dioxide in patients with tracheal intubation during general anesthesia, However, many factors may possibly affect the accuracy of P_et_CO_2_ monitoring, such as mismatch of ventilation to blood flow (V/Q ratio), chronic obstructive pulmonary disease, obstructive sleep apnea syndrome, surgery postures, smoking, ect. Recently, noninvasive transcutaneous carbon dioxide partial pressure (P_TC_CO_2_) monitoring has been used in infants and in adult patients with good accuracy [1]–[3].

Laparoscopic bariatric surgery is a quite common operation for the treatment of severe obese patients. Griffin J et al [4] reported that the P_TC_CO_2_ monitoring had a better accuracy than that of P_et_CO_2_ in estimate of P_a_CO_2_ for sever obesity undergoing open bariatric surgery, but the accuracy and correlation between P_a_CO_2_ measurements and P_TC_CO_2_ monitoring for patients with laparoscopic bariatric surgery is still unknown. We therefore designed the present study to evaluate the accuracy and correlation of estimating P_a_CO_2_ using a P_TC_CO_2_ monitor in severe obese patients undergoing laparoscopic bariatric surgery.

## Materials and Methods

### Ethics Statement

This study was approved by the ethic committee of Jiangsu Province Hospital. Before the study, oral informed consent was obtained from each participant. The Ethics Committee of Jiangsu Province Hospital approved oral informed consent because the study was to be of minimal risk.

We consulted with the Ethics Committee of Jiangsu Province Hospital before the experiment and were confirmed that the P_TC_CO_2_ monitor was capable of monitoring the CO_2_ level non-invasively. Meanwhile, it was almost impossible to cause thermal injuries on the skin or skin allergy, which in turn made it a non-invasive medical instrument. So the monitor could be applied to the selected patients as long as their oral consents were obtained in the first place. We recorded the conversation of inquiries of those consents by note and were fully supported by the Ethics Committee.

### Data

22 patients were collected from our hospital, who were ASA I–II and scheduled for laparoscopic bariatric surgery. Patients with history of severe trauma, operations, smoking, and severe cardiovascular or respiratory diseases, such as coronary heart disease, congestive heart failure, or chronic obstructive pulmonary disease were excluded from this study.

Anesthesia was induced with propofol (1–2 mg.kg^−1^), fentanyl (2–4 μg.kg ^−1^), and rocuronium (0.6 mg.kg^−1^) by the same anesthetist. After tracheal intubation, patients were ventilated with 100% oxygen (2 L/min) under the mode of intermittent positive pressure ventilation (IPPV), with a tidal volume of 6–10 ml/kg and an I:E ratio of 1∶2. The ventilatory frequency and tidal volume were adjusted to maintain normocarbia (P_et_CO_2_, 35–45 mmHg). The P_et_CO_2_ was monitored by side stream spirpometry (Datex-Ohmeda, Finland, air pumping speed 150 ml.min^−1^). P_TC_CO_2_ was monitored with a TCM-4 device (Radiometer, Copenhagen, Denmark). One of the authors calibrated, placed, and maintained the monitor. Before placement, the electrode was cleaned, a new membrane applied, and calibration done according to the manufacturer’s recommendations. The working temperature of the electrode was set at 44°C and the electrode was placed on the chest. The area where the electrode was placed was swabbed with alcohol in order to to facilitate adhesion of the disk to the skin. Re-calibration was required once the position of the electrode was changed. The electrode was removed, adjusted, and replaced in a different location on the chest every 2 h to avoid thermal injury.

An AS/5 monitor (Datex-Ohmeda, Finland) was employed to monitor the patients’ electrocardiography, pulse oxymetry, and noninvasive blood pressure. Before anesthesia, patients’ heart rate (HR) and arterial blood pressure were both recorded as baseline. A 20-G or 22-G arterial catheter was inserted into the left radial artery under local anesthesia for ABG sampling. P_a_CO_2_ from the ABG was determined with an i-STAT Analyzer System with Disposable EG4-cartridges. Before ABG sampling was performed, the patients’ blood pressure, HR, tidal volume, and respiratory rate were constant for at least 5 minutes to obtain the stable P_TC_CO_2_. P_TC_CO_2_ and P_et_CO_2_ were recorded simultaneously. Anesthesia was maintained with propofol (5–8 mg.kg^−1^.h^−1^), remifentanil (0.1–0.2 μg kg^−1^.min^−1^), and atracurium (0.6 mg. kg^−1^.h^−1^) to keep the variation of blood pressure and HR within 20% of baseline values. Those patients whose blood pressure drop was more than 20% baseline value or who needed a vasoconstrictor to maintain hemodynamics stable were excluded from the study. yet the data from them before hypotension occurred could still be used for the analysis. The patient’s body temperature was continuously monitored nasopharyngeally and maintained at 36°C to 37°C. The room temperature was maintained at 23°C to 25°C. Pneumoperitoneum was established and intraperitoneal CO_2_ infusion pressure was maintained at 12–14 mmHg during the surgery.

### Statistic

Data were presented as mean±SD. Statistical analysis was performed by SPSS version 17.0(SPSS Inc, USA).We assessed the agreement between P_a_CO_2_ and P_TC_CO_2_ or P_a_CO_2_ and P_et_CO_2_ using Bland–Altman method. Pearson correlation coefficient and linear regression analysis were used to establish the relationship. The exact probability method and two way contingency table were employed to compare the difference of 5 mmHg or less and 3 mmHg or less between P_a_CO_2_ and the other two noninvasive variables. A P value of 0.05 or less was considered statistically significant.

## Results

**Figure 1:**
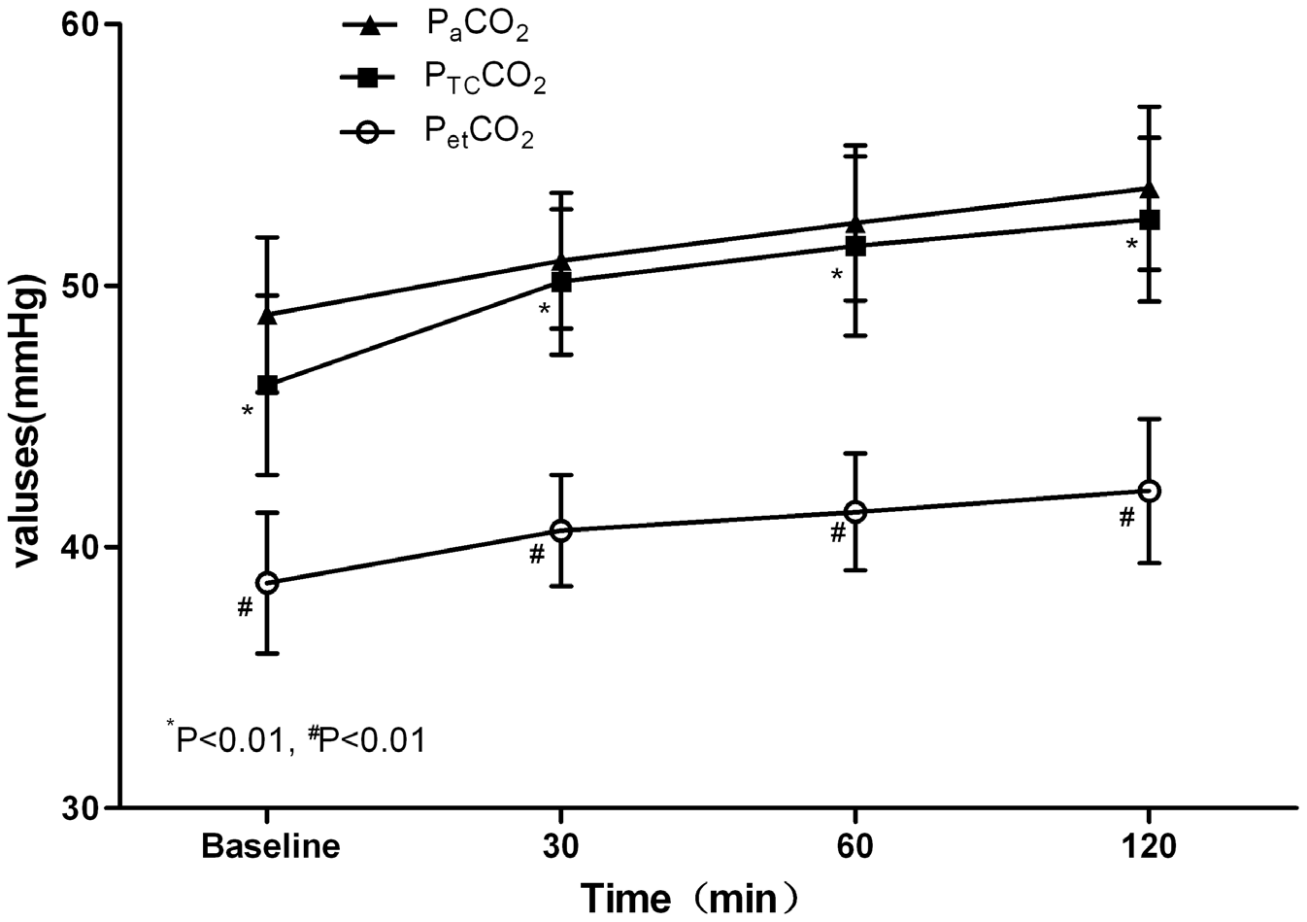
P_et_CO_2_, P_TC_CO_2_ and P_a_CO_2_ at different time points after CO_2_ pneumoperitoneum. End tidal carbon dioxide partial pressure (P_et_CO_2_), transcutaneous carbon dioxide partial pressure (P_TC_CO_2_), and arterial carbon dioxide partial pressure (P_a_CO_2_) at baseline, 30 minutes after, 60 minutes after, and 120 minutes after CO_2_ pneumoperitoneum. ^*^P<0.01, compared with P_a_CO_2_. ^#^P<0.01, compared with P_a_CO_2_.

**Figure 2:**
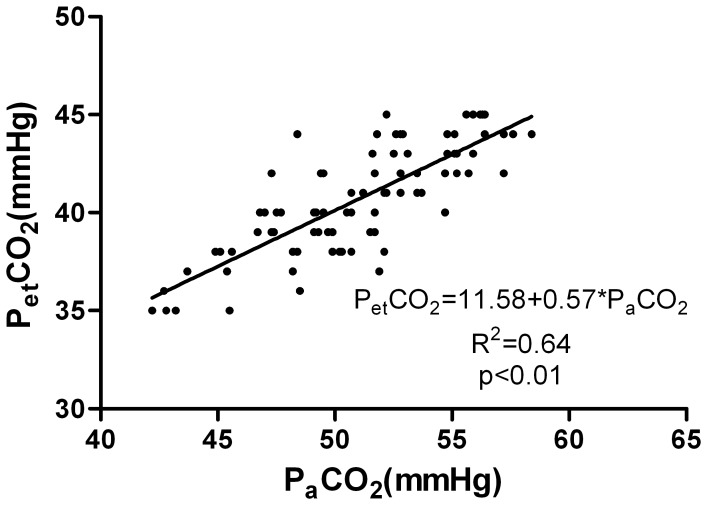
Linear regression analysis between P_et_CO_2_ and P_a_CO_2_. Linear regression analysis between the end tidal carbon dioxide partial pressure (P_et_CO_2_) and the arterial carbon dioxide partial pressure (P_a_CO_2_) in 21 severe obese patients during laparoscopic bariatric surgery. The linear regression equation: P_et_CO_2_ = 11.58+0.57×P_a_CO_2_, r^2^ = 0.64, P<0.01.

**Figure 3:**
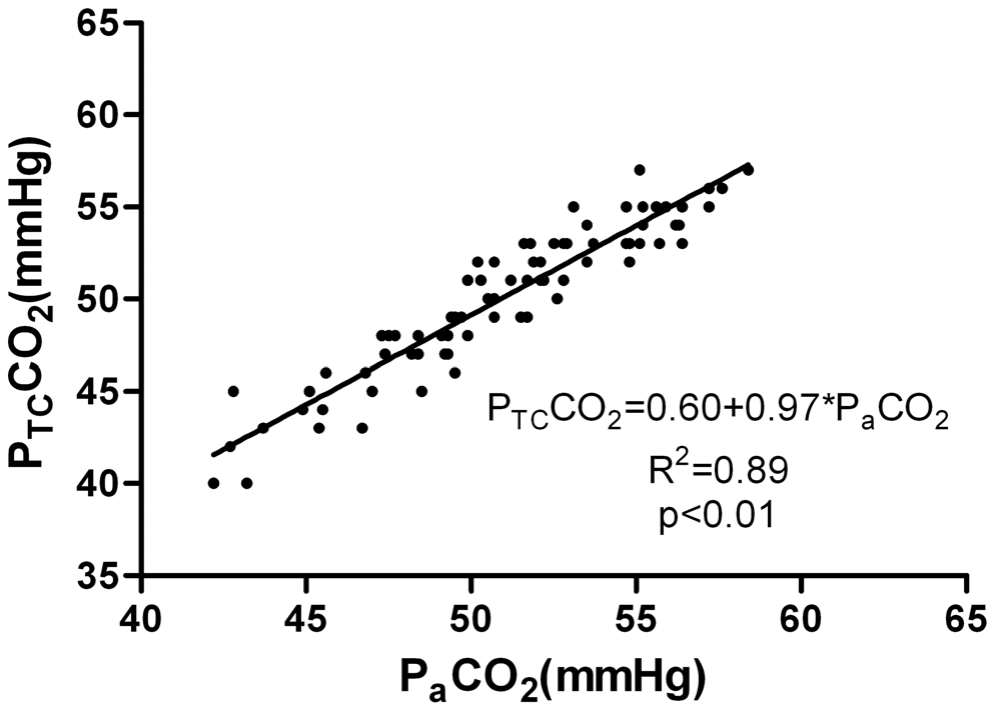
Linear regression analysis between P_TC_CO_2_ and P_a_CO_2_. Linear regression analysis between the transcutaneous carbon dioxide partial pressure (P_TC_CO_2_) and the arterial carbon dioxide partial pressure (P_a_CO_2_) in 21 severe obese patients during laparoscopic bariatric surgery. The linear regression equation: P_TC_CO_2_ = 0.60+0.97×P_a_CO_2_, r^2^ = 0.89, P<0.01.

**Figure 4:**
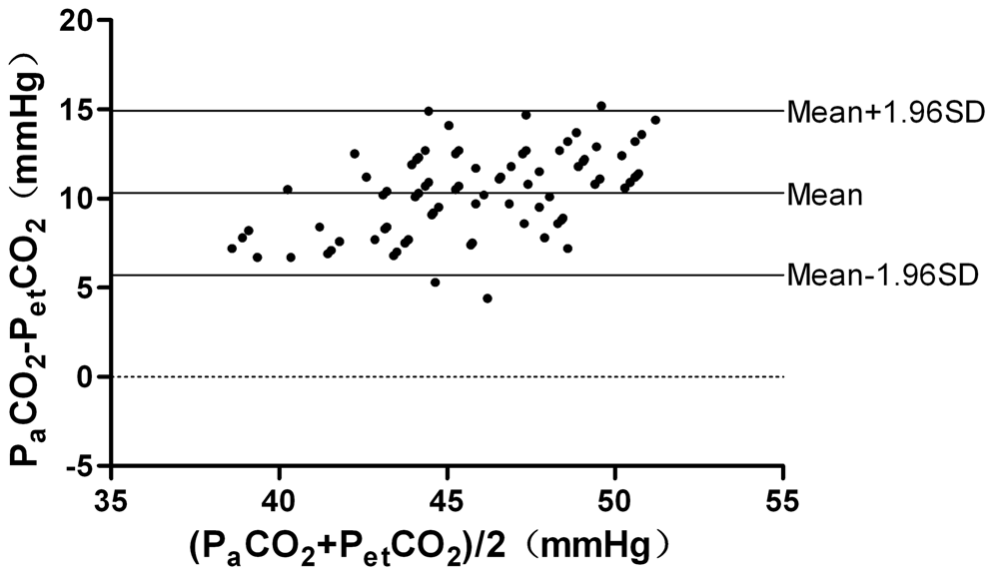
Agreement between P_et_CO_2_ and P_a_CO_2_. Agreement between P_et_CO_2_ and P_a_CO_2_ by the Bland-Altman method. Plot of the arterial carbon dioxide minus the end tidal carbon dioxide (y-axis) against the mean of the end tidal carbon dioxide and the arterial carbon dioxide (x-axis).The bias and precision are labeled. According to the Bland-Altman analysis, the 95% limits of agreement (LOA) of the average P_a_CO_2_–P_et_CO_2_ difference was 10.3±4.6 mmHg (mean±1.96 SD).

**Figure 5:**
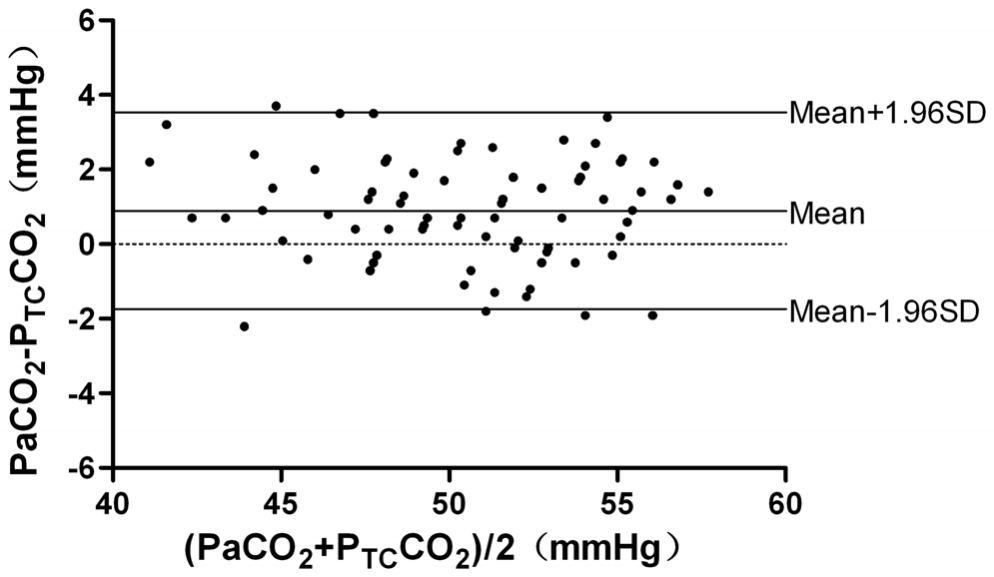
Agreement between P_TC_CO_2_ and P_a_CO_2_. Agreement between P_TC_CO_2_ and P_a_CO_2_ by the Bland-Altman method. Plot of the arterial carbon dioxide minus the transcutaneous carbon dioxide (y-axis) against the mean of the transcutaneous carbon dioxide and the arterial carbon dioxide (x-axis).The bias and precision are labeled. The 95% limits of agreement (LOA) of the average P_a_CO_2_–P_TC_CO_2_ difference was 0.9±2.6 mmHg (mean±1.96 SD).

21 patients (8 men and 13 women; age from 19–55 yr, 29(9)yr; weight from 86 to 160 kg, 119.3(22.1)kg; BMI from 35.3 to 51.1 kg/m^2^, 42.1(5.4) kg/m^2^) were recruited into this study. All patients underwent laparoscopic bariatric surgery. The P_a_CO_2_, P_et_CO_2_, and P_TC_CO_2_ values were recorded at 4 time points. Eight-four samples were finally obtained. The mean values of these variables at different time points are presented in Table 1 and Figure 1. In these samples, P_TC_CO_2_ was correlated with P_a_CO_2_ at each time point (r = 0.90, 0.89, 0.93 and 0.90, respectively, P<0.01). P_et_CO_2_ was correlated with P_a_CO_2_ at each time point (r = 0.66, 0.71, 0.69 and 0.86, respectively, P<0.01). The P_a_CO_2_ values were ranging from 42.2 to 58.4 mmHg. The average P_a_CO_2_–P_et_CO_2_ difference was 10.3±2.3 mmHg and the average P_a_CO_2_–P_TC_CO_2_ difference was 0.9±1.3 mmHg. In those samples, both P_et_CO_2_ and P_TC_CO_2_ were closely correlated with P_a_CO_2_. The linear regression equation between P_et_CO_2_ and P_a_CO_2_ was P_et_CO_2_ = 11.58+0.57×P_a_CO_2_, r^2^ = 0.64, P<0.01(Figure 2); and P_TC_CO_2_ and P_a_CO_2_ was P_TC_CO_2_ = 0.60+0.97×P_a_CO_2_, r^2^ = 0.89, P<0.01(Figure 3). In all samples, there wasn’t a difference of 3 mmHg or less between P_a_CO_2_ and P_et_CO_2_, yet there was a difference of 3 mmHg or less between P_a_CO_2_ and P_TC_CO_2_ in 79 of the 84 samples (P<0.01). Only one P_et_CO_2_–P_a_CO_2_ difference (absolute value) was 5 mmHg or less while all values of P_TC_CO_2_–P_a_CO_2_ difference (absolute value) were 5 mmHg or less (P<0.01). According to Bland-Altman analysis, the 95% limits of agreement (LOA) of the average P_a_CO_2_–P_et_CO_2_ difference was 10.3±4.6 mmHg (mean±1.96 SD, Figure 4), while the 95% limits of agreement (LOA) of the average P_a_CO_2_–P_TC_CO_2_ difference was 0.9±2.6 mmHg (mean±1.96 SD, Figure 5).

**Table 1:** CO_2_ Partial Pressure at Different Time Points (Mean±SD, n = 21).

Before pneumoperitoneum			After pneumoperitoneum					
	Baseline (mmHg)	r	30 min (mmHg)	r	60 min (mmHg)	r	120 min (mmHg)	r
PaCO_2_	46.89±2.97		50.95±2.59		52.40±2.97		53.73±3.12	
PetCO_2_	38.62±2.69	0.66	40.62±2.13	0.71	41.33±2.24	0.69	42.14±2.76	0.86
P_TC_CO_2_	46.19±3.43	0.90	50.14±2.78	0.89	51.52±3.44	0.93	52.52±3.14	0.90

Correlation between end-tidal carbon dioxide partial pressure (P_et_CO_2_) and arterial carbon dioxide partial pressure (P_a_CO_2_) and transcutaneous carbon dioxide partial pressure (P_TC_CO_2_) and P_a_CO_2_ at 4 time points. P<0.01.

## Discussion

The end-tidal carbon dioxide (P_et_CO_2_) measurement has been widely used in the anesthetic management. However, quite a number of factors may possibly affect the accuracy of P_et_CO_2_ measurement, including alveolar ventilation volume, V/Q ratio, chronic obstructive pulmonary disease, etc. Due to the effects of weight loss, remission of diabetes mellitus and improvement of health-related quality, bariatric surgery gets rapid development in clinical practice [5]. For those patients with severe obesity, the functional residual capacity (FRC) is reduced with increase of intrapulmonary shunt (10–25%), especially for those with abdominal obesity. And the CO_2_ pneumoperitoneum results in the further decrease of FRC and greater degree of intrapulmonary shunt, which diminishes the accuracy of P_et_CO_2_. Nevertheless, P_TC_CO_2_ monitoring uses heated electrodes to improve local perfusion (capillary arterialisation), which facilitates the absorption of carbon dioxide into the heated electrodes via skin diffusion. The carbon dioxide inside the electrodes changes the internal pH value, which results in P_TC_CO_2_ signals. Reid CW et al [6] reported that the P_a_CO_2_–P_et_CO_2_ difference increased along with P_a_CO_2_ levels. In the patients undergoing bariatric surgery the P_et_CO_2_ is usually greater than 40 mmHg, and P_a_CO_2_ may be underestimated, while the P_TC_CO_2_ is still accurately reflect the P_a_CO_2_ levels. Especially after the extubation, P_et_CO_2_ monitor becomes unavailable. And it is highly likely that hypoxia and hypercapnia happens. P_TC_CO_2_ monitoring is particularly valuable. In our study, the average P_a_CO_2_–P_et_CO_2_ difference was 10.3±2.3 mmHg whereas the average P_a_CO_2_–P_TC_CO_2_ difference was 0.9±1.3 mmHg. Those findings are unanimous with the past study.

Previous studies reported that the CO_2_ partial pressure was at its highest 30 minutes after pneumoperitoneum and was stable 60 minutes after pneumoperitoneum [7]–[9]. But Cuevlier et al [10] suggested that O_2_ partial pressure was stable 5 minutes and CO_2_ partial pressure got stable 20 minutes after pneumoperitoneum, resulting from accumulation of more CO_2_ in vivo. Therefore, determination of P_TC_CO_2_ 30 minutes after pneumoperitoneum in our study was feasible for most patients. Xue Q et al [11] suggested that in prolonged laparoscopic surgery P_TC_CO_2_ monitor was more accurate than P_et_CO_2_, and the linear regression equations were P_TC_CO_2_ = 0.74×P_a_CO_2_+11.07, r^2^ = 0.71, P<0.0001; P_et_CO_2_ = 1.04×P_a_CO_2_+6.45, r^2^ = 0.55, P<0.01. However, the correlation of the P_TC_CO_2_ and P_a_CO_2_ is unknown more than 60 minutes after pneumoperitoneum. In this study, the laparoscopy was applied during the whole surgery process, and we found P_TC_CO_2_ and P_a_CO_2_ still demonstrated excellent correlation 120 minutes after pneumoperitoneum (r = 0.93).

Griffin J et al [4] found that carbon dioxide monitoring by using P_TC_CO_2_ was more accurate in patients with a BMI greater than 40 kg/m^2^ undergoing transabdominal bariatric surgery. Maniscalco M et al [12] suggested that in patients (BMI, 43.7 kg/m^2^) with chronic obstructive pulmonary disease (COPD), obstructive sleep apnea syndrome (OSAS), hypopnea syndrome (OHS) and respiratory failure (RF), P_TC_CO_2_ still accurately reflected the P_a_CO_2_,compared with the blood gas analysis. Our findings indicated that P_TC_CO_2_ was more accurate in reflecting the real levels of P_a_CO_2_ than P_et_CO_2_ in patients with BMI>35 kg/m^2^ undergoing laparoscopic bariatric surgery. And only one P_et_CO_2_ readings was 5 mmHg or less from P_a_CO_2_ while all values of P_TC_CO_2_ were 5 mmHg or less.

The successful P_TC_CO_2_ monitoring depends on monitor and a series of patient factors. Although TC-CO_2_ monitoring more accurately reflected P_a_CO_2_ in most of the patients in the previous studies, several technical factors may affect the accuracy of monitor, including trapped air bubbles, improper placement, damaged membranes, and inappropriate calibration techniques. Patient-related factors may also affect the accuracy, such as variations in skin thickness, the presence of edema, tissue hypoperfusion, or the use of vasoconstricting drugs and oxygen deficiency acidosis. Nishiyama T et al [13] found P_TC_CO_2_ and P_TC_O_2_ more precisely predict the P_a_CO_2_ and P_a_O_2_ when the electrodes were put on the chest, compared to the placement at the upper arm and forearm. Nishiyama T etc [14] found chest electrode was better than the ear electrode. We put the electrode on the left side chest (between nipple and clavicle), which was easy to be observe by the anesthesiologists and reduced the influence of electrode caused by the body movement during abdominal operation.

Electrode heating temperature can significantly affect the accuracy of the measurement results. Nishiyama T et al [15] suggested that the electrode should be heated to at least 43°C in adults patients, when P_TC_CO_2_ and P_TC_O_2_ could accurately estimate P_a_CO_2_ and P_a_O_2_ respectively. Sorensen LC et al [16] found that lower electrode temperature increased systematic error of measurements in premature and newborns. The higher the electrode temperature was, the greater the risk of burn injury was.Accordingly, in our study we set the electrode temperature at 44°C as manufactures recommedation, and no patient suffered from postoperative skin burn, while skin erythema occurred in 15 patients, and disappeared in 24 hours.

Skin tissue perfusion also affected the accuracy of the P_TC_CO_2_ monitoring. Lower environmental temperature resulted in skin vascular contraction, reduced blood flow, In this study temperature in the operating room always maintained above 23°C. Meanwhile, patients’ exposure was reduced to the lowest possible level. Currently there are still disputes about the accuracy of P_TC_CO_2_ monitoring with the use of vasopressors, and past studies suggested vasopressors affected accuracy of P_TC_CO_2_ monitoring [16], [17], whereas Berkenbosch JW [2] and Rodriguez P [18] found that vasopressors didn’t affect the accuracy of the P_TC_CO_2_ monitoring. Most of general anesthetics have the effect of vasodilation. Propofol administration produced venodilation and peripheral vasodilation in humans [19]. Opioids (like fentanyl and remifentanil) produced concentration-dependent and endothelium-independent relaxations in human being radial artery rings [20]. In our study only one patient received vasoconstrictor during anesthesia induction and the other 21 patients not.

We all know that during jet ventilation (JV), P_et_CO_2_ may underestimate P_a_CO_2_ because of inadequate washout of the anatomical dead space by a small tidal volume and the relatively slow response time of infrared CO_2_ analyzers. Especially during use of high frequency jet ventilation (HFJV) [21]. But the transcutaneous devices provide an effective method for non-invasive monitoring of P_a_CO_2_ in situations where continuous and precise control of CO_2_ levels in perioperative with HFJV [22]. P_TC_CO_2_ monitoring especially useful during use of HFJV in obese patients which can avoid them in high risks of hypercapnia.

However, P_TC_CO_2_ can not substitute the P_et_CO_2_ monitoring completely as P_et_CO_2_ monitoring has many unique advantages, including the judgment of successful intubation, the warning of breathing circuit disconnected and indicator of pulmonary embolism, etc. In addition, the P_et_CO_2_ wave pattern had more clinical significance. The P_TC_CO_2_ monitoring was limited by many factors, including longer priming time, adjuscting before use, periodically electrode replacement, no CO_2_ waveform and the risk of skin burn injury [23].

## Conclusion

In conclusion, our study demonstrated that P_TC_CO_2_ can estimate the P_a_CO_2_ more accurately than P_et_CO_2_ in obese patients under laparoscopic bariatric surgery. Moreover, the application of P_TC_CO_2_ monitoring might improve the quality of the anesthesia management.
